# Propagation network of tailings dam failure risk and the identification of key hazards

**DOI:** 10.1038/s41598-022-08282-1

**Published:** 2022-04-02

**Authors:** Zhixin Zhen, Xu Wu, Bo Ma, Huijie Zhao, Ying Zhang

**Affiliations:** 1 School of Civil and Resource Engineering, University of Science and Technology Beijing, Beijing, 100083 China; 2 Beijing Municipal Engineering Research Institute, Beijing, 100037 China

**Keywords:** Statistical physics, thermodynamics and nonlinear dynamics, Engineering

## Abstract

The tailings dam system is complex, and the dam structure changes continuously over time, which can make it difficult to identify key hazards of failure and characterize the accident formation process. To solve the above problems, based on complex network theory, the paper uses the identified hazards and the relationship between hazards to construct the propagation network of tailings dam failure risk (PNTDFR). The traditional analysis methods of network centrality usually focus on one aspect of the information of the network, while it cannot take into account to absorb the advantages of different methods, resulting in the difference between identified key nodes and real key hazards. To find the key hazards of tailing dam failure, based on the characteristics of multi-stage propagation of failure risk, the paper proposes a multi-stage collaborative hazard remediation method (MCHRM) to determine the importance of hazard nodes by absorbing the advantages of different centrality methods under different hazard remediation (deletion) ratios. The paper applies the above methods to Feijão Dam I. It can be found that when the priority remediation range is increased to 45%, the key hazards obtained by the MCHRM will cover all the causes of accidents proposed by the Dam I failure investigation expert group. Besides, the paper compares the monitoring data, daily inspection results and safety evaluation information of key hazards with the ‘Grading standards of hazard indicators’, and obtains the formation process of the Dam I failure and 30 key hazards in trigger state.

## Introduction

The composition of tailings is very complex, which may show strong corrosive, volatile, acidic and other characteristics affected by the types of minerals mined. If the tailings can not be managed effectively, the tailings may leak under the tailings dam failure, which will pose a serious threat to the surrounding environment and communities. On January 25, 2019, the Feijão Dam I in Brazil suddenly broke. More than 200 people died or were missing in the tailings dam accident. The Dam I has a complete management system and monitoring system, using ground-based radar, satellite (InSAR), high-definition video and drones and other advanced monitoring equipment, but before the accident, it was not found that the tailings dam had significant abnormal signals that may cause a failure^1,2^. This shows that even in tailings dams with a very high level of safety management, there are still some key accident hazards that have not been discovered or effectively monitored. Therefore, the use of effective methods to timely and accurately to identify the key hazards in the tailings dam system, and to control the various hazards that induce accidents in the bud or latent state, is of great significance for preventing accidents and reducing the risks of tailings dam failure.

The identification of hazards and the determination of their characteristics are an important part of system safety management. It not only defines the scope of research for subsequent accident analysis and prevention, and post-disaster rescue, but also provides decision basis for managers. There are dozens of commonly used methods for identifying hazards, such as failure type and impact analysis, pre-hazard analysis, checklist method, hazard and operability research, fault tree analysis, event tree analysis^3^. In response to the differences in research systems, scholars have proposed a series of new hazard identification methods that are more suitable for the research system based on these conventional hazard identification methods. According to the results of accident analysis and interviews, Nascimento F et al. applied grounded theory and template analysis to compile a list of hazards affecting pilots’ night flight capabilities^4^. With the help of safety specialists' experiences, Alizadehsalehi S et al. used BIM software used in the design of the structure to identify potential safety hazards in buildings^5^. Chen RC et al. passed a multivariate Cox regression analysis and a nomogram model to identify potential hazards related to the fatal outcome of COVID-19^6^.

In the research on the identification of hazards in tailings dam, scholars have done a lot of research work. Based on the e-EcoRisk database, Rico M et al. analyzed 147 cases of tailing pond accidents around the world, and found 15 reasons for tailing dam failure^7^. Li Zhaodong et al. established a checklist of factors affecting the tailings dam accidents, and assigned points to it^3^. Pier-Luc Labonté-Raymond et al. have studied the impact of climate change on the drainage system of tailings ponds^8^. MG Lemos et al. identified the chemical, mineralogical and metallurgical properties of gold tailings located in the Santa Barbara mine^9^. Baker K E et al. applied process safety management tools to the tailings storage and transportation system, and visually characterized the possible hazards and control measures to prevent accidents^10^. The safety management of tailings dam is a whole-process management, and the hazards are coupled and influenced by each other during the whole life cycle. Therefore, the above methods are difficult to complete and systematically identify the hazards of tailings dams. In order to overcome these problems, facing the life cycle of tailings ponds and combining the four influencing factors of natural factors, design factors, construction factors and management factors, Zhao Yiqing et al. proposed the process-causing grid method to identify hazards of tailings ponds. Although the process-causing grid method can identify the hazards of tailings dams relatively completely and systematically, it relies more on the subjective judgment of researchers, and the supporting evidence for the identification of hazards is not clear^11^.

Complex network can well characterize the internal relationship between research objects(nodes)^12^, and therefore, it has been widely used in many fields in recent years^13–18^. Most complex networks are scale-free, and a small number of hub nodes play a leading role in the operation of the network^19^. In other words, if we find the key nodes in the network and attack them, the normal operation of the network will be disrupted. In order to identify the key nodes in a complex network, Yu E Y et al. generated a feature matrix for each node in the network, and used a convolutional neural network to train and predict the influence of the node^20^. Hou B et al. used the all-around distance method to find influential nodes in complex networks^21^. AXZ et al. used the information transfer probability between any pair of nodes and the k-medoid clustering algorithm to identify influential nodes in complex networks with community structure^22^. Freeman LC etc. defined centrality in terms of the degree to which a point falls on the shortest path between others^23^. In order to rank the spreaders, an average shortest path centrality is proposed^24^. Qin Xuan et al. applied the centrality analysis of the complex network to the study on the important hazards of tailings pond accidents^25^. At present, although some scholars have begun to try to introduce complex network theory into the identification of key accident hazards, they mainly use several commonly used complex network centrality indicators, and have not made corresponding improvements according to different application objects.

The key hazards obtained based on the complex network theory do not consider the severity of the hazards, and these hazards may be different from the real accident hazards. If these hazards are evaluated and graded, the actual impact of these hazards on the accident can be determined. The evaluation and classification methods of hazards are mostly safety evaluation methods. Wu Qi et al. firstly established an assessment index system of leakage accident risk, and then used the analytic hierarchy process and the fuzzy comprehensive evaluation method to quantify the influencing factors of the accident risk, and finally calculated the hazards level^26^. Shi Zongbao et al. have redefined safety hazards and put forward a more reasonable classification standard for safety hazards^27,28^. In the process of risk assessment, Zhao Dongfeng et al. used the consequences of accidents to approximate the consequences of hazards, and solved the problem of risk classification of specific hazards^29^. Tta B et al. used epigenetic biomarkers as a tool to assess chemical hazards^30^. However, in the risk management of tailings dams, no scholars have yet to determine the status of key accident hazards by grading hazard level, so as to find out the true formation process of dam failure.

In order to solve the above problems, the paper proposes a three-dimensional hazard identification framework (THIF) to identify the hazards and propagation paths of failure risk in a tailings dam. Then, the complex network theory is used to establish a propagation network of tailings dam failure risk (PNTDFR) that is universal for the entire industry, and some important network characteristics are analyzed. To find the key hazards (nodes) in the PNTDFR, the paper will absorb the advantages of different centrality indicators under different hazard governance (node deletion) ratios, and study the new network centrality method. After importance of hazards is confirmed, it can be confirmed that those key hazards caused the dam break by confirming the trigger state of the key hazards. Finally, the above method is applied to the Feijão Dam I to verify the accuracy.

## Research method

### 1Hazard identification and network establishment

The ‘hazard’ is the potential occurrence of an event within a prescribed time and space, and its definition has been expanded as a process, phenomenon or human activity^31^. In order to avoid the subjectivity of hazard identification, this paper proposes a new hazard identification method from the perspective of safe production: a three-dimensional hazard identification framework (THIF). This method selects accident cases, laws and regulations, standard specifications, documents and other materials as evidence for hazard identification, and systematically identify the hazards of the personnel, material, environment, and management in tailings dams based on the life cycle of the construction, operation, closure, and reclamation of tailings dams^32^.

The paper uses the identified hazards of tailings dams and the evolution relationship between hazards to construct an adjacency matrix, and then import the adjacency matrix into Pajek software, and construct a propagation network of tailings dam failure risk (PNTDFR). The nodes in the PNTDFR represent hazards, and the edges represent the relationship between hazards. According to the status change of the hazards, the PNTDFR is divided into three layers of hazard nodes (dormant hazard, armed hazard, activity hazard or accident) and two stages (from dormant hazard to armed hazard, from armed hazard to activity hazard)^32,33^. The initial dormant hazards can only cause other hazards and cannot be caused by other hazards. The in-degree value of dormant hazards is 0, including all initial nodes of the four influencing factors of tailings dam failure, such as floods, excessive rainfall, and excessive standard earthquakes. Armed hazards are formed by the evolution of the dormant hazards or other armed hazards, and these armed hazards will may cause damage accidents under certain working environments or conditions, such as the rapid rise of pond water level, the dam deformation, and the tailings liquefaction. These hazards mean the imminent accidents and disasters. Active hazards are accidents that are or have occurred. If these active hazards cannot be effectively suppressed, they will lead to serious consequences and disasters, including overtopping and dam break and so on^24^.

When the network model is established, we can use complex network theory to analyze the statistical features of the PNTDFR, such as degree, betweenness centrality, network density, characteristic path length and clustering coefficient. From these characteristics, the propagation law of tailings dam failure risk can be analyzed and discovered.

### Analysis of key hazard nodes

When the PNTDFR is a scale-free network, the PNTDFR will appear vulnerable to deliberate attacks^34^. In other words, if we can prioritize to remedy the hazard nodes that have a greater impact on network connectivity, the propagation efficiency of the network can be reduced, thereby slowing down or even blocking the propagation of risks. Therefore, the paper chooses network efficiency as an index to measure the spreading ability of dam-break risk.

Global network efficiency, also known as network connectivity, refers to the difficulty of average network connectivity, which is the average of the sum of the reciprocal lengths of the shortest path between all pairs of hazard nodes in the entire network^34^. Degree centrality, betweenness centrality and closeness centrality are commonly used methods to characterize the importance of nodes in complex networks. In this paper, the importance of hazard nodes determined by the three methods is used as the priority of hazard remediation (node deletion), and then the differences of the three methods in reducing network efficiency are compared. By absorbing the advantages of different methods under different hazard remediation ratios, combined with the characteristics of multi-stage propagation of tailings dam failure risk, this paper proposes a multi-stage collaborative hazard remediation method (MCHRM) to determine the importance of hazard nodes. The specific implementation process of this method is as follows:

1. Since the first-layer nodes (dormant hazards) only have out-degree values, and the betweenness centrality is 0, only the degree value needs to be considered in determining the remediation order of the first-layer hazards, and priority is given to the hazard nodes with greater degree value.
2. The second-layer nodes (armed hazards) have degree values, betweenness centrality and closeness centrality, which are in the intermediate stage between the dormant hazard and the activity hazard. Therefore, it is necessary to consider the influence of three indicators on risk propagation at the same time. When there are differences among three hazard remediation methods under different remediation proportions, priority is given to the remediation method that can reduce the speed of risk evolution faster.
3. The third-layer nodes (activity hazards) are the possible accident modes of a tailings dam, and the remediation method is the same as that of the second-layer node. The hazard of dam break is the object of the accident studied in this paper, so it is not remedied.
4. After the remediation priority of hazards at the same layer according to the corresponding methods is determined, those nodes with a smaller remediation proportion will be prioritized among hazard nodes at different layers.

When all the hazard nodes of the PNTDFR have been treated, by observing the change trend of network efficiency, the key hazard nodes of the PNTDFR can be determined (those nodes that can significantly reduce network efficiency after deleting). In this paper, these important nodes are called key hazards in failure accident. In addition, if the MCHRM can reduce network efficiency more effectively than the commonly used methods in the past, the remediation (deletion) order of hazards (nodes) determined by the MCHRM can better characterize the importance of hazards in the dam failure accident.

### Accident formation process

If you want to determine which of the hazards caused an accident, you need to determine whether these hazards are in a triggered state and how serious. Because the China Tailings Pond Safety Grade Classification Standard divides the tailings ponds into four levels: normal, mild, moderate, and dangerous, the paper also divides the grading standards of the key hazard indicators of tailings dams into four levels combining the Technical Regulations for Safety of Tailings Pond and the Code for Design of Tailings Facilities. Level 1 is a normal state, level 2 is a mild danger, level 3 is a moderate danger, and level 4 is a serious danger. In the classification of grading standards, the indicators that can obtain specific values are classified using quantitative analysis methods. For example, the evaluation indicator of hazard 5 (Heavy rainfall) is rainfall, which is calculated in the depth of the water layer per unit area within 24 h. Hazards that are difficult to quantitatively classify are qualitatively used. For example, hazard 355 (Insufficient experience in personnel or organization qualification problems) are divided into four levels based on the personnel’s education, working hours, and qualification levels of the institution. According to the above method, the paper has formulated the grading standards of some hazard indicators, as shown in “Appendix B”.

When the classification standard of the key hazard is completed, by comparing the monitoring data, daily inspection results, and safety evaluation information before the accident, the levels of the key hazards in the studied tailings dam can be obtained, so as to determine the states of these hazards^35^. The key hazards of level 1 are in a normal state and will not further evolve or cause other hazards. These hazards are not the key hazards that causes the tailings dam to break. The remaining hazards with a level greater than 1 are the key hazards that led to the dam failure. By excluding the hazard nodes of level 1, we can determine the key hazards and evolution paths between hazards. In the accident investigation report, these key hazards are also referred to as the main cause of the accident.

## Case analysis

The paper takes Feijão Dam I in Brazil as a case. The crest elevation of Dam I is 942 m, the maximum height is 86 m, and the dam crest length is 720 m. The height of each sub-dam varies from 5 to 18 m. The slope of the upstream and downstream slopes is between 1:2.5 and 1:1.5, and the other slopes of the dam body generally adopt a slope of 1:2. After 2013, Dam I stopped the construction of tailings dam. Later, in July 2016, the stockpiling of tailings was stopped and the tailings pond was closed. More information about Dam I can be found in Report of the Expert Panel on the Technical Causes of the Failure of Dam I^36^.

### Identification of hazards and the relationship between Hazards

A total of 117 hazards and 535 relationships are obtained by the THIF method, as shown in “Appendix A”^32^. In “Appendix A”, the first column indicates the categories of hazards, including four categories: environment factor, personnel factor, material factor, and management factor. The second column indicates the number (ID) of the hazards in the third column. The fourth column indicates the number of the hazards caused by the hazard in the third column. For example, the hazard named ‘heavy rainfall’ in the second row of the third column is numbered 5, which belongs to the environment factor. Through the THIF method, we can get the hazards that may be caused by the ‘heavy rainfall’. These hazards are numbered 19, 67, 69, 150, 193 and 19.

### Propagation network of Dam I failure risk

**Figure 1:**
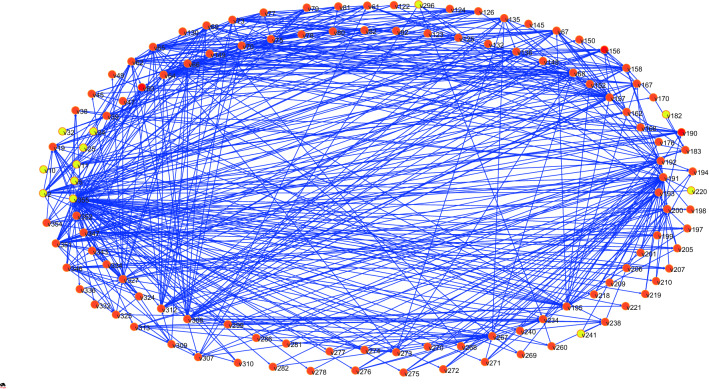
Mode of the I-FRPN.

This section uses hazards of Dam I and the relationship between the hazards in “Appendix A” to construct the adjacency matrix, and then import it into Pajek software to construct the propagation network of Dam I failure risk (I-FRPN), as shown in Fig. 1.

#### Degree and degree distribution

**Figure 2:**
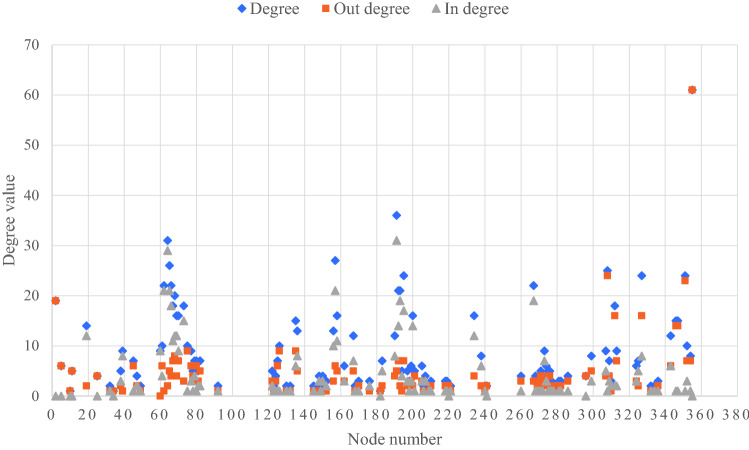
Node degree in the I-FRPN.

The degree value of each node in I-FRPN can be obtained through Pajek complex network software as shown in Fig. 2. The average degree of the I-FRPN is 9.15, and the network density is 0.04, indicating that a hazard node is directly related to 9.15 hazard nodes on average, but the overall density of the I-FRPN is not large.

It can be seen from Fig. 2 that among the top 10 hazards, 355 (Insufficient experience in personnel or organization qualification problems) is the hazard node with the largest degree value in the I-FRPN, which directly affects 61 hazards. It shows that if the personnel and organization do not have sufficient experience or do not meet the corresponding qualification requirements, the tailings dam will always be threatened throughout its life cycle. 191 (Fracture of drainage structure) is directly related to 36 hazards, which is the second largest hazard in the degree value. It is classified as a material factor among the four influencing factors. The degree values of 62 (partial landslide and collapse of the dam), 64 (Dam instability), 65 (Dam deformation), 157 (Filter failure), 195 (Rapid rise of pond water level) and 327 (Safety monitoring facilities cannot fully reflect the operating status of the tailings pond) are respectively 22, 31, 26, 27, 24 and 24. These hazards belong to the material factor together with the hazard 191, and account for 70% of the top 10 hazards, highlighting the fact that the material factor plays a leading role in tailings dam safety management.

Hazard 308 (Closure design not in accordance with regulations) has a degree value of 25, which belongs to the same personnel factor as hazard 355, and these hazards are indirect factors that lead to dam break. 351 (Improper maintenance) is directly related to 24 hazards, which is the management factor, indicating that management plays an important role in the safety management of tailings dams.

From the point of view of out degree, the values of hazards 355 (Insufficient experience in personnel or organization qualification problems), 308 (Closure design not in accordance with regulations), 351 (Improper maintenance), 2 (Flood) and 312 (Dam body remediation does not meet the requirements) are respectively 61, 24, 23, 19, and 16, which are the five nodes with the largest out-degree value, indicating that personnel factors, management factors, and environmental factors are more likely to cause other hazards. 191 (fracture of drainage structure), 62 (partial landslide and collapse of dam), 64 (dam instability), 65 (dam deformation), and 157 (failure of water filter body) are the 5 hazards with the highest in-degree value, and in-degree values are respectively 31, 29, 21, 21, and 21. These hazards all are material factors, indicating that material factors are prone to form armed hazards under the influence of dormant hazards.

**Figure 3:**
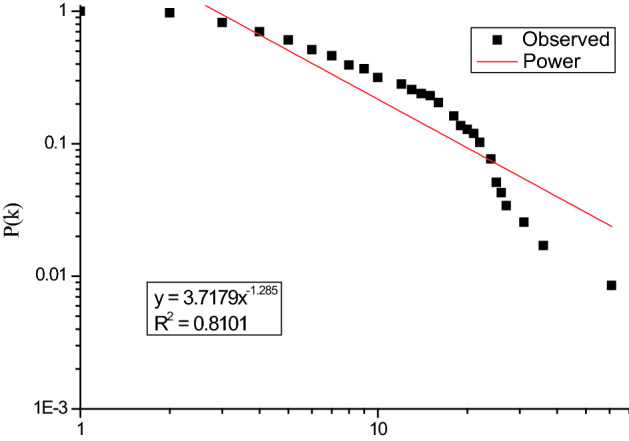
Cumulative degree distribution of the I-FRPN.

Cumulative degree distribution of the I-FRPN is shown in Fig. 3. The cumulative degree distribution presents a power-law distribution that has the approximate fit $P\left(k\right)={3.7179x}^{-1.285}$ (${R}^{2}=0.8101$). The above result deviates from the power-law nature for lager k, which indicates that the I-FRPN has scale-free property^18,37^. It means that a few hub nodes play a dominant role in the I-FRPN. If we can find these key nodes, the spread of risk can be slowed down or even blocked, thus preventing the occurrence of dam break. The degree studied in this section is an important indicator for judging the importance of network nodes. In addition, there are also indicators such as betweenness centrality and closeness centrality that are also commonly used to measure the importance of nodes. In the next section, we will conduct more analysis on this aspect.

#### Network diameter and average path length

The network diameter, also known as the maximum path length of a network, represents the largest step length between two nodes in the network^34^. After calculation, the network diameter of the I-FRPN is 8, which means that a hazard node can affect any node in the network only after a maximum of 8 steps. The most distant node pairs of the network are v32 and v150 or v7 and v45. Compared with some accident networks studied in the past^15,37,38^, the diameter of I-FRPN is larger, and the evolution path of the risk is complicated.

The characteristic path length is also called the average path length. After calculation, the average path length of the I-FRPN is 2.81, indicating that it takes less than 3 steps on average to transfer the risk of dam break from one hazard to another hazard. The above results show that the characteristic path length of the I-FRPN is small, and the risk of dam break can be spread quickly on the network. If no corresponding measures are taken, the emergence of a serious hazard may cause a tailings dam break in a relatively short time.

#### Clustering coefficient and small-world property

**Figure 4:**
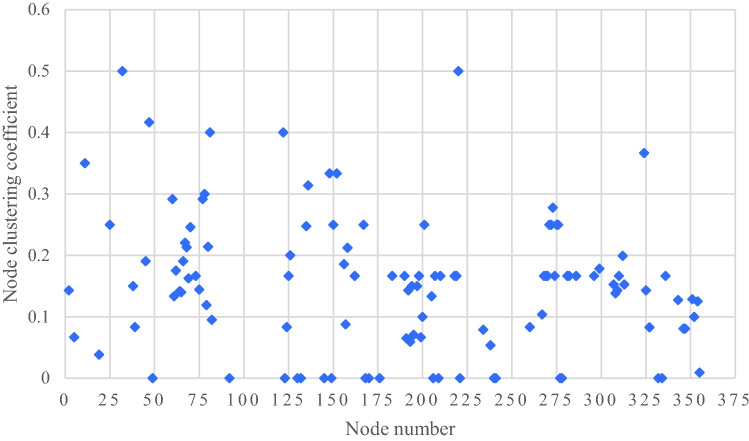
Clustering coefficient of nodes in the I-FRPN.

The clustering coefficient of the I-FRPN refers to the degree of interconnection between adjacent nodes of a hazard node in the network^37^. That is to say, there is no clustering coefficient for nodes with a degree value of 1. In this paper, the average clustering coefficient of the I-FRPN is calculated by Pajek software as 0.15. After excluding the nodes with a degree of 1, the clustering coefficients of the hazard nodes in the network are obtained, as shown in Fig. 4. It can be seen from the figure that the clustering coefficient of the hazard node in the I-FRPN is between 0 and 0.5. The clustering coefficients of hazard 32 (Insufficient tank length) and 220 (The maximum flow rate of flood control structure design is greater than the allowable flow rate of building materials) are both 0.5, which are the nodes with the largest clustering coefficient, indicating that the adjacent hazards of the hazard 32 and 220 have a strong correlation and show strong clustering.

**Figure 5:**
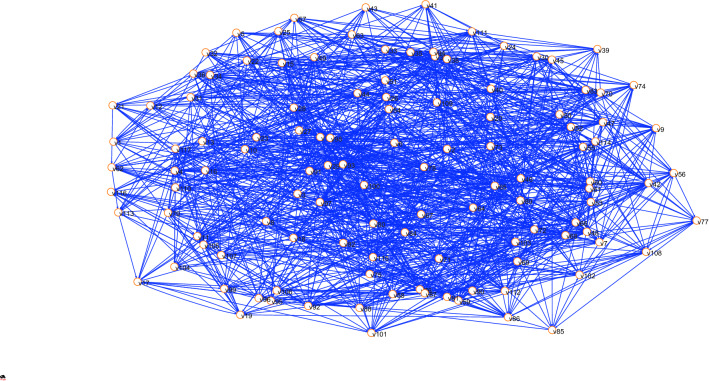
Mode of WW.

Small-world networks usually have large clustering coefficients and small characteristic path lengths^34^. In order to judge whether the clustering coefficient of the I-FRPN meets the requirements of the small world, this paper constructs a random network with the same number of nodes and the same degree value as the I-FRPN, and calculates the clustering coefficient to be 0.08, which is smaller than the clustering coefficient of the I-FRPN (0.15). The equal-sized dam failure risk random network is called the mode of WW, as shown in Fig. 5. Combined with the characteristic path length of the I-FRPN is only 2.81, it can be concluded that the I-FRPN has small-world property. In other words, the break accident for Dam I has the characteristics of multi-factor coupling and short disaster path.

### Key hazards nodes in the I-FRPN

**Figure 6:**
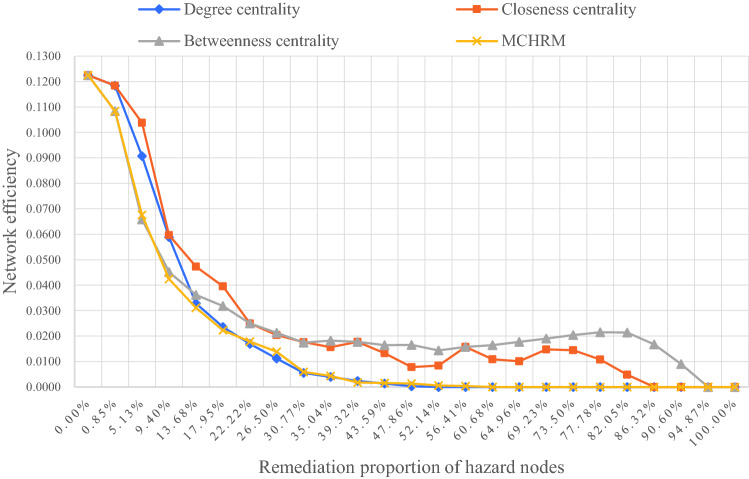
The impact of the four remediation sequences of hazard nodes on network efficiency.

The paper first treats(deletes) the node with the largest index value and calculates the network efficiency, and then calculates the network efficiency after every 5 hazard nodes are treated. Figure 6 shows the changes of the network efficiency under the different hazard remediation methods.

In Fig. 6, it can be found that the preferential treatment of nodes with large betweenness centrality can achieve better results in the early stage (low deleting proportion). In other words, when the remediation proportion of hazard nodes is small, the risk propagation speed can be reduced more quickly by the betweenness centrality. However, when the proportion of hazard remediation reaches 13.68%, the hazard node with a higher degree value will have a better effect of reducing risk spread.

It can be seen from Fig. 6 that the MCHRM performs better than the other three commonly used methods, whether in the early stage of the hazard node remediation or in other stages. In addition, we can also find that all four methods show that when the proportion of node remediation reaches about 30%, the decline in network efficiency tends to slow down significantly. Further increasing the proportion of node remediation will not significantly reduce the propagation efficiency of the network. In other words, when we are in the process of hazard remediation of tailings dams, if we give priority to the top 30% of hazard nodes determined by the MCHRM, we can use the vulnerability of the network to reduce network efficiency more quickly. In this paper, these hazard nodes that can quickly reduce the propagation efficiency of the I-FRPN are called key hazards of Dam I failure, and the relationships between these key hazards are called the key propagation paths.

### Failure process of Feijão Dam I

Since the weights between nodes in I-FRPN are assumed to be equal, only relying on network centrality analysis may miss the key hazards of Dam I failure. In order to overcome the problem, the paper has expanded the selection range of key hazards and set the key hazards as the top 45% of the index value. The I-FRPN has a total of 117 hazard nodes, and the top 45% of the index value includes 53 nodes. The expanded key hazards determined by MCHRM are shown in Table 1. The first column of Table 1 is the serial number of the key hazards, indicating the importance of the hazards. The third column is the name of the hazard to be studied, the second column is the number (ID) of the hazard, the sixth column is the level of the corresponding hazard node, and the fourth and fifth columns are the degree value and the betweenness centrality of the hazard node.

**Table 1:** Key hazards of Dam I failure.

Sequence number	Node number	Node name	degree centrality	betweenness centrality	Hazard level
1	195	Rapid rise of pond water level	24	0.05123	2
2	64	Dam instability	31	0.0035	3
3	65	Dam deformation	26	0.0356	3
4	157	Filter failure	27	0.0370	3
5	191	Fracture of drainage structure	36	0.0457	2
6	192	Leaking drainage structure	21	0.0299	2
7	158	Leakage channel	16	0.0284	3
8	267	Pipes and grooves deformation	22	0.0243	1
9	308	Closure design not in accordance with regulations	25	0.0004	1
10	327	Safety monitoring facilities cannot fully reflect the operating status of the tailings pond	24	0.0223	3
11	355	Insufficient experience in personnel or organization qualification problems	61	0.0000	2
12	62	Local landslide and collapse of the dam	22	0.0014	3
13	68	Uneven settlement of the dam	20	0.0199	3
14	66	Dam crack	22	0.0145	2
15	69	Scour the dam	16	0.0185	1
16	351	Improper maintenance	24	0.0003	1
17	67	Dam surface water saturation	18	0.0139	3
18	70	Tailings liquefaction	16	0.0123	4
19	193	Scour or cavitation drainage structures	21	0.0094	1
20	200	Insufficient flood discharge capacity	16	0.0101	3
21	234	Blockage or siltation	16	0.0166	2
22	2	Flood	19	0.0000	1
23	73	Poor stability of tailings dam slope	18	0.0074	3
24	136	Dam foundation instability	13	0.0080	3
25	238	Serious corrosion of equipment	8	0.0090	1
26	312	Dam body renovation does not meet the requirements	18	0.0017	1
27	5	Heavy rainfall	6	0.0000	3
28	39	Insufficient storage capacity of tailings pond	9	0.0074	1
29	135	Uneven foundation subsidence	15	0.0062	1
30	167	Seepage line is higher than control seepage line	12	0.0076	2
31	325	Monitoring instrument failure, work interruption	7	0.0073	1
32	19	Landslides in the tailings pond	14	0.0061	1
33	61	Poor control of tailings deposits	10	0.0041	1
34	343	Inadequate safety evaluation	12	0.0033	1
35	346	Improper data management	15	0.0000	1
36	347	Insufficient or wrong hydrological and geological data	15	0.0000	1
37	45	Tailings particle size/gradation does not meet the requirements	7	0.0023	2
38	75	Improper calculation method of tailings dam stability	10	0.0003	2
39	183	Filter failure	7	0.0031	2
40	273	Subsidence or deformation of supporting facilities such as pipes, trenches and tunnels	9	0.0029	1
41	11	Liquefied soil, soft clay and collapsible loess foundation	5	0.0000	2
42	156	Leakage damage	13	0.0016	4
43	190	Overtopping	12	0.0128	1
44	126	Unreasonable design of cast-in-place protective surface	10	0.0001	1
45	307	Pump failure	9	0.0017	1
46	352	Design defects of emergency plan	10	0.0006	3
47	47	Excessive tailings unit weight	4	0.0012	4
48	49	Strongly corrosive tailings	2	0.0010	1
49	77	The tailings dam slope ratio is unreasonable	9	0.0009	3
50	309	Close the tailings pond without understanding the hazards and risks	7	0.0010	1
51	313	The improvement of flood discharge system does not meet the requirements	9	0.0013	2
52	82	The dam layout is unreasonable (the location sub dam and primary dam)	7	0.0008	3
53	25	There are mining activities near the site	4	0.0000	1

By consulting the monitoring data, daily inspection results and safety evaluation information of each hazard before the failure of Dam I, the level of each hazard is obtained according to the grading standards of hazard indicators in “Appendix B”, as shown in Table 1.

**Figure 7:**
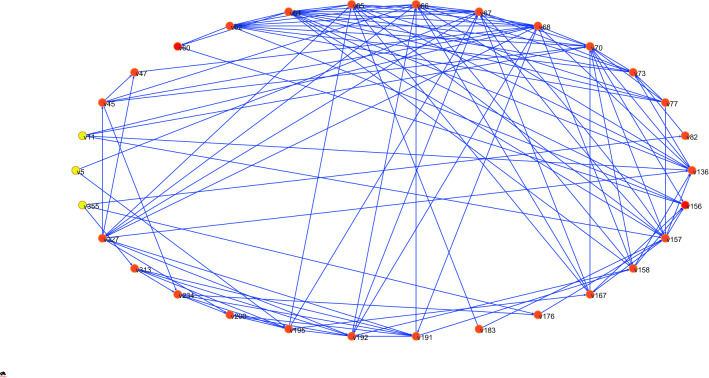
Key hazards and propagation paths of Dam I failure.

By excluding the hazard nodes of level 1 in the normal state, we can determine that there are 30 key hazards in the failure accident of Feijão Dam I. Combining the evolution relationship among the hazards based on evidence in “Appendix A”, we can obtain the 240 propagation paths between key hazards. The key hazards and propagation paths of Dam I failure are shown in Fig. 7.

## Comparison and analysis

To verify whether the key hazards (causes) of the Dam I failure identified are reasonable, this paper compares the above results with the conclusions made by the accident investigation expert group chaired by Dr. Peter K. Robertson. The expert group concluded that the direct cause of the failure of the Dam I was the tailings liquefaction of the dam. The expert group conducted research on the composition of the dam body material and the dam-break trigger mechanism, and found that 6 technical problems were the main causes leading to the dam break. Compare the key hazards with a level greater than 1 in Table 2 with the main causes found by the expert group^36^, as shown in Table 2.

**Table 2:** Key hazard comparison table.

Technical problem	Node number	Node name
(1) A design that resulted in a steep upstream constructed slope	77	The tailings dam slope ratio is unreasonable
(2) Water management within the tailings impoundment that at times allowed ponded water to get close to the crest of the dam, resulting in the deposition of weak tailings near the crest	195	Rapid rise of pond water level
(3) A setback in the design that pushed the upper portions of the slope over weaker fine tailings	82	The dam layout is unreasonable
(4) A lack of significant internal drainage that resulted in a persistently high water level in the dam, particularly in the toe region	200/167	Insufficient flood discharge capacity / Seepage line is higher than control seepage line
(5) High iron content, resulting in heavy tailings with bonding between particles. This bonding created stiff tailings that were potentially very brittle if triggered to become undrained	47	Excessive tailings unit weight
(6) High and intense regional wet season rainfall that can result in significant loss of suction, producing a small loss of strength in the unsaturated materials above the water level	5/70	Heavy rainfall / Tailings liquefaction

Through comparison, it can be found that the main causes of the Dam I failure proposed by the expert group are 6 aspects, involving 8 hazard nodes. When the key hazards identified by the MCHRM are used as the priority remediation criteria (top 30%), the hazard 5, 70, 167, 195, and 200 are consistent with the causes of the dam failure mentioned in the expert group’s conclusion, accounting for 62.5% of the 8 hazards; when the priority remediation range is increased to the top 45%, hazard 47, 77 and 82 are also included.

The above comparison results show that the MCHRM can better find the key causes of the dam failure. When the priority remediation range is increased by 15%, it will be possible to cover all the main causes. Although the conclusions of the expert group cannot be completely equated with the true causes and risk propagation paths of Dam I failure, the expert group members have rich experience and outstanding academic attainments on the issue of tailing dam failure. Therefore, expert group’s conclusion is highly reliable. In addition, the failure causes and risk propagation paths of the Dam I identified in the paper also involve some hazards and propagation paths that the expert group did not mention, which may include some problems that the expert group did not notice, so it will help improve the safety management of tailings dams.

## Discussion

**Figure 8:**
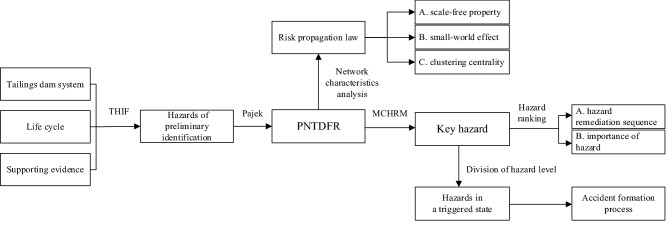
A flow chart showing the full-text research methods and results.

The research content of the paper mainly includes hazard identification, the construction of the propagation network of tailings dam failure risk (PNTDFR), the analysis of the law of risk evolution, the identification of key hazards of tailings dam failure, and the characterization of the accident formation process. A flow chart showing the full-text research methods and results is shown in Fig. 8.

The paper analyzes the laws and regulations, technical specifications and procedures related to tailings dams one by one, and refers to relevant scientific and technological literature, accident cases and other supplementary evidence, to identify the hazards that may exist in the different life cycle stages of the tailings dam system. The method is called a three-dimensional hazard identification framework (THIF). This method not only avoids the omission of hazards, but is also more objective than the subjective identification methods in the past.

Compared with commonly used methods such as accident trees and accident chains, complex networks can more completely and systematically link these evidence-based hazards, and characterize the evolution process of dam-break risk in the form of a network. In addition, through the analysis of the network characteristics of I-FRPN, it is found that the propagation of dam-break risk presents a small-world and scale-free nature, while the distribution of hazards shows clustering features. The above characteristics have not been discovered in the conventional failure analysis of tailings dams.

In the determination of key hazards, we can see from Fig. 6 that the commonly used degree centrality, betweenness centrality, and close centrality can all indicate the importance of the hazard node to a certain extent, because the network efficiency all showed a rapid decline after hazard nodes with big indicator value are deleted. The hazards with greater betweenness centrality bear more risk propagation tasks, while the closeness centrality of hazards reflects its connection with other hazards. The two centrality indicators reflect the importance of hazards from different points. The MCHRM not only utilizes the advantages of the three indicators, but also combines the characteristics of multi-stage propagation of dam-break risk, which better reflects the importance of hazards of dam-break. For the problem of hazard remediation, the importance of hazards determined by MCHRM also represents the best order of hazard remediation.

The MCHRM can significantly reduce the network efficiency, but there are also the problems that the severity or level of the hazards is not considered, and the weights between nodes in the network are assumed to be equal, which will lead to a certain difference between identified key hazards and real key hazards from tailings dam failure. At the same time, due to the complex causes of dam failure accidents and the difficulty of quantification, it is difficult to accurately give the weight of the relationship between hazards. In order to solve the above problems, this paper sets a certain reserve range when determining the range of key nodes in the PNTDFR, that is, increases the range of priority remediation. The specific reserve range can be adjusted to a certain extent according to the difference of the research objects.

Key hazards are the main causes of dam failure, but for a specific accident, not all key hazards have played an important role. Therefore, if you want to reproduce the process of the accident, you must determine the status of these key hazards in the risk evolution of the accident. The network constituted by key hazards in the trigger state and the relationship between hazards intuitively represents the whole process of the accident.

Although this paper has done a lot of work to find the key hazards and characterize the accident formation process, there are still three shortcomings: a. Although the reserve range of priority remediation can cover all key hazards of dam failure, it is difficult to give an accurate reserve ratio, and the actual application needs to combine the experience of some technical personnel. b. In the formulation of hazard grading standards, due to the numerous influencing factors of hazards and the difficulty of quantifying some of the influencing factors, the grading standards of some hazards adopt a subjective qualitative classification method, which affects the accuracy of some grading indicators. c. The hazards and the relationships between hazards in this paper are all based on evidence (accident cases, laws and regulations, documents and media, etc.), but the reliability of different evidences is different, which will affect the accuracy of the research. To better solve the above problems and improve the practicability of above methods, the author of this paper plans to study more accident cases in the next step, so as to determine a more specific reserve range of priority remediation and build a hazard information database of tailings dams, which are suitable for the whole industry. At the same time, the paper will consider the evidence according to the reliability of the evidence, and select more quantitative indicators to classify the hazard indicators to improve the practicability of the methods.

## Conclusion

The paper proposes an identification method: a three-dimensional hazard identification framework (THIF), which can identify the hazards of tailings dam accidents in a more systematic, complete and objective manner. Applying it to Dam I, based on the life cycle stage, dam structure, surrounding environment, personnel composition, and management system of the tailings dam, it is found that there may be 117 hazards and 535 relationships between hazards in this tailings dam. Based on the identified hazards and the relationship between hazards, this paper uses hazards to represent nodes and the relationship between hazards to represent edges, and constructs an I-FRPN that characterizes the propagation process of Dam I failure risk. Through the analysis of characteristics, it is found that the propagation of the failure risk of Dam I presents a small-world and scale-free effect.

By absorbing the advantages of betweenness centrality and degree centrality under different remediation proportion of hazard nodes in finding key hazard nodes, the paper proposes the MCHRM to identify the key hazards and the priority remediation order among the key hazards combined with the three-layer and two-stage characteristics of the PNTDFR. By analyzing the I-FRPN, it can be found that when the priority remediation range is increased from 30 to 45%, the key hazards obtained by the MCHRM will cover all the causes of accidents proposed by the Dam I failure investigation expert group. At the same time, the paper compares the monitoring data, daily inspection results and safety evaluation information of key hazards with the "Grading standards of hazard indicators", confirms that 30 key hazards are in trigger state, and obtains the formation process of the Dam I failure.

## Appendix A: List of hazards of tailings dam failure

**Table:**

Impact factors	Number(v)	Name of hazards or factors	Number of hazards caused
Environment factor	2	Flood	19, 60, 62, 64, 65, 66, 67, 69, 150, 156, 158, 167, 190, 191, 192, 193, 195, 273, 325
	5	Heavy rainfall	19, 67, 69, 150, 193, 195
	10	Gravel foundation	157
	11	Liquefied soil, soft clay and collapsible loess foundation	68, 70, 135–136, 157
	19	Landslides in the tailings pond	39, 195
	25	There are mining activities near the site	19, 62, 64, 66
	32	Insufficient impoundment length (upstream wet tailings impoundment)	39
	34	Large catchment area	195
Material factor	39	Insufficient storage capacity of tailings pond	190
	45	Tailings particle size/gradation does not meet the requirements	47, 66, 68, 61, 234
	47	Excessive tailings unit weight	68, 61
	49	Strongly corrosive tailings	238
	60	Dam break	
	62	Local landslide and collapse of the dam	60
	64	Dam instability	60, 62
	65	Dam deformation	62, 64, 157, 267, 273
	66	Dam crack	62, 64, 73, 158
	67	Dam surface water saturation	62, 64–66, 73, 157
	68	Uneven settlement of the dam	62, 64–66, 191–192, 267, 273
	69	Scour the dam	62, 64–66
	70	Tailings liquefaction	62, 64, 68, 136, 156–158
	73	Poor stability of tailings dam slope	62, 64
	77	The tailings dam slope ratio is unreasonable	62, 64–65, 73, 157
	78	Unreasonable width of dam crest	62, 64–65, 157
	79	Improper dam type selection for the initial dam	39, 64, 157
	80	The height of initial dam is unreasonable	39, 64–65, 73, 81, 194
	81	The ratio of the initial dam height to the total dam height of the upstream tailings dam is unreasonable	64–65, 73
	82	The dam layout is unreasonable (the location sub dam and primary dam)	32, 39, 69, 73, 135
	61	Poor control of tailings deposits	64–65, 68, 77, 152, 157
	92	The tailings dam is not equipped with anti-scouring measures	69, 82
	122	There is a horizontal weld on the slope	64, 66, 73
	132	No effective filter layer is set on the dam foundation	157
	135	Uneven foundation subsidence	66, 68, 73, 136, 191, 267, 273
	136	Dam foundation instability	64–66, 68, 73
	149	The length or thickness of the horizontal paving in front of the dam is insufficient	157
	150	Natural paving (covering) is destroyed	158
	152	Poor deposition control for dry beach face	157
	156	Leakage damage	60, 62, 64
	157	Filter failure	64, 67, 136, 156, 167, 195
	158	Leakage channel	64, 68, 135–136, 156
	167	Seepage line is higher than control seepage line	65–67, 156
	176	Poor drainage of composite geotechnical drainage network	157
	182	Unqualified filter material	183
	183	Filter failure	65, 157
	190	Overtopping	60, 62, 64, 69
	191	Fracture of drainage structure	66, 69, 158, 192, 200
	192	Leaking drainage structure	66–67, 69, 150, 158, 195, 200
	193	Scour or cavitation drainage structures	191–192
	194	Insufficient regulating water storage	39
	195	Rapid rise of pond water level	39, 65, 67, 152, 167, 190, 194
	197	The foundation pit at the higher groundwater level has no drainage facilities	195, 200
	198	The flood drainage system does not match the dam construction method	191, 200
	200	Insufficient flood discharge capacity	193, 195
	206	Insufficient elevation of drainage holes in front of the dam	200
	207	Flood drainage structures are directly located on the tailings sediment beach	191
	209	Insufficient foundation bearing capacity of underground flood drainage structures	191
	210	Improper installation of flood interception and drainage facilities	191, 200
	218	Improper installation of energy dissipation facilities	191, 193
	219	No energy dissipation measures have been taken in the tailings facility	191, 193
	220	The maximum flow rate of flood is greater than the allowable flow rate of the building materials	191, 193
	221	The clarified water of the tailings pond is not used for backwater utilization	195
	234	Blockage or siltation	176, 191, 195, 200
	238	Serious corrosion of equipment	191, 325
	240	No anti-corrosion treatment in tailings facilities	238
	241	Unqualified anti-corrosion materials	193, 238
	260	Improper handling of local hydraulic phenomena	234, 238, 267
	267	Pipes and grooves deformation	191, 193, 234
	268	Defects of the interception ring in pipe body	69, 192–193
	269	The pipe body is in direct contact with the big rocks	191, 267
	270	The outer wall of the pipe is not protected	191, 267
	271	The dimensions of pipes, grooves, tunnels, etc. do not meet the requirements	191, 193, 234, 267
	272	Pipes and grooves material unqualified	191, 193, 267
	273	Subsidence or deformation of supporting facilities such as pipes, trenches and tunnels	191, 267
	275	Excessive slope deviation for laying pipes, trenches, tunnels, etc	191, 193, 234, 239, 267
	276	Improper design of corners of pipes, grooves, tunnels, etc	191, 193, 234, 267
	277	Improper subgrade design of Pipes and grooves	193, 234
	278	Improper design of slope ratio of pipe trench and embankment	193
	281	Poor quality of fill around the pipeline	191, 267
	282	The axial filling height of the pipe in the dam body is different	191, 267
	286	The joint length of the drain pipe is unreasonable	191–192, 267
	296	Poor pump quality	192–193, 234, 307
	299	Improper installation of pump	192, 195, 200, 234, 307
	307	Pump failure	61, 100, 127, 192, 195, 200, 231
	310	The surrounding environment improvement does not meet the requirements	19
	312	Dam body renovation does not meet the requirements	62, 64–70, 73, 135–136, 148, 157–158, 167, 183
	313	The improvement of flood discharge system does not meet the requirements;	191–192, 195, 234, 267, 273, 307
	325	Monitoring instrument failure, work interruption	327, 343
	327	Safety monitoring facilities cannot fully reflect the operating status of the tailings pond	19, 45, 47, 49, 65–69, 135–136, 191–192, 200, 267, 343
	334	The number of water quality monitoring wells around the tailings pond is insufficient	327
Management factor	168	Improper measures to reduce the seepage line	167
	170	Insufficient protection measures for seepage prevention facilities	158, 183
	336	The setting of monitoring facilities is not included in the construction plan	325, 327
	343	Inadequate safety evaluation	19, 60, 156, 190, 200, 327
	346	Improper data management	38, 79, 162, 197, 199, 205, 207, 274, 299, 309, 324, 327, 343, 352
	347	Insufficient or wrong hydrological and geological data	38, 79, 162, 197, 199, 205, 207, 274, 299, 309, 324, 327, 343, 352
	351	Improper maintenance	60, 62, 64–70, 156–158, 167, 183, 190–193, 234, 238, 267, 307, 325
	352	Design defects of emergency plan	19, 60, 62, 156, 190–191, 195
	354	Insufficient emergency plan drills	19, 60, 62, 156, 190–191, 195
Personnel factor	38	Inaccurate storage capacity calculation	39, 194
	75	Improper calculation method of tailings dam stability	64, 73, 77–81, 92
	123	Improper selection and care of slope protection turf	73
	124	Slope cutting did not follow the design requirements	19, 64–65
	125	Slope protection was not carried out in time	19, 62, 64–65, 73, 122
	126	Unreasonable design of cast-in-place protective surface	19, 62, 64–66, 73, 77, 122, 157
	145	No coverage measures in the pond area	157
	148	Weakness of paving has not been reinforced	158
	130	Poor construction quality of horizontal paving	157
	162	Unreasonable anti-seepage design	19, 156–157
	199	The determination of the flood control standard of the tailing pond is not accurate	190, 194, 200
	201	Blocking defects of flood drainage facilities	192–193, 195, 200
	205	The installation location and elevation of drainage facilities do not meet the design requirements	193, 195, 200
	274	Improper installation of supporting facilities	191, 267, 273
	308	Closure design not in accordance with regulations	19, 62, 64–70, 73, 135–136, 148, 157–158, 167, 183, 191–192, 234, 238, 267, 273, 307
	309	Close the tailings pond without understanding the hidden dangers and risks	66, 310, 312–313
	324	Improper selection of monitoring instruments and equipment	327, 343
	332	No monitoring of groundwater and surrounding water bodies	327
	355	Insufficient experience in personnel or organization qualification problems	31, 38, 75, 79, 82, 61, 123–134, 145, 148–149, 162, 168, 170, 176, 197–199, 201, 205–207, 209–210, 218–221, 240, 260, 268–272, 274–278, 281–282, 286, 299, 308–310, 312–313, 324, 332, 334, 336, 343, 346–347, 351–352, 354

## Appendix B: Grading standards of hazard indicators

**Table:**

Grading indicator	Classification and value of grading indicator			
	1	2	3	4
Personnel experience or organization qualification	> 0.75	0.50–0.75	0.25–0.50	< 0.25
Flood (once in N years)	< 5	5–20	20–50	> 50
Rainfall (mm/24 h)	< 50	50–100	100–200	> 200
Liquefaction degree of tailings	< 0.25	0.25–0.50	0.50–0.75	> 0.75
The degree of impact of mining activities near the pond area	< 0.25	0.25–0.50	0.50–0.75	> 0.75
The height from the warning water level (m)	> 8.00	8.00–4.00	4.00–0.00	< 0.00
Fracture degree of drainage structure	< 0.25	0.25–0.50	0.50–0.75	> 0.75
Water leakage degree of drainage structure	< 0.25	0.25–0.50	0.50–0.75	> 0.75
Deformation degree of dam	< 0.25	0.25–0.50	0.50–0.75	> 0.75
Deformation degree of Pipe (groove)	< 0.25	0.25–0.50	0.50–0.75	> 0.75
Leakage channel	< 0.25	0.25–0.50	0.50–0.75	> 0.75
Dam settlement	< 0.25	0.25–0.50	0.50–0.75	> 0.75
Filter body	> 0.75	0.50–0.75	0.25–0.50	< 0.25
Monitoring blind spots of safety monitoring facilities	< 0.25	0.25–0.50	0.50–0.75	> 0.75
Blockage or siltation	< 0.25	0.25–0.50	0.50–0.75	> 0.75
Scoured dam	< 0.25	0.25–0.50	0.50–0.75	> 0.75
Dam crack	< 0.25	0.25–0.50	0.50–0.75	> 0.75
Water content of the dam surface	< 0.25	0.25–0.50	0.50–0.75	> 0.75
Dam foundation stability	< 0.25	0.25–0.50	0.50–0.75	> 0.75
Flood discharge capacity	> 0.75	0.50–0.75	0.25–0.50	< 0.25
Degree of erosion or cavitation of drainage structures	< 0.25	0.25–0.50	0.50–0.75	> 0.75
Equipment corrosion degree	< 0.25	0.25–0.50	0.50–0.75	> 0.75
Height of seepage line (m)	> 8.00	6.00–8.00	1.40–1.70	< 1.40
Remaining storage capacity of tailings pond	> 60%	20–60%	10–20%	< 10%
Monitoring instrument stability	> 0.75	0.50–0.75	0.25–0.50	< 0.25
Slope stability of tailings dam	> 0.75	0.50–0.75	0.25–0.50	< 0.25
Degree of foundation subsidence	< 0.25	0.25–0.50	0.50–0.75	> 0.75
Possibility of landslides in the pond area	< 0.25	0.25–0.50	0.50–0.75	> 0.75
Sedimentation level of tailings	> 0.75	0.50–0.75	0.25–0.50	< 0.25
Dam stability	> 0.75	0.50–0.75	0.25–0.50	< 0.25
Calculation method of dam stability	> 0.75	0.50–0.75	0.25–0.50	< 0.25
Design of dam surface protection	> 0.75	0.50–0.75	0.25–0.50	< 0.25
Completeness and accuracy of information	> 0.75	0.50–0.75	0.25–0.50	< 0.25
Maintenance log	> 0.75	0.50–0.75	0.25–0.50	< 0.25
Emergency plan	> 0.75	0.50–0.75	0.25–0.50	< 0.25
Safety assessment	> 0.75	–	–	0
Status of supporting facilities such as pipes, trenches, tunnels, etc	> 0.75	0.50–0.75	0.25–0.50	< 0.25
Filter	> 0.75	0.50–0.75	0.25–0.50	< 0.25
Tailing particle size	> 0.50	0.20–0.50	0.05–0.20	< 0.05
Pump	> 0.75	0.50–0.75	0.25–0.50	< 0.25
Renovation of the dam body	> 0.75	0.50–0.75	0.25–0.50	< 0.25
The degree of local landslide and collapse of the dam	< 0.25	0.25–0.50	0.50–0.75	> 0.75
Flood drainage system renovation	> 0.75	0.50–0.75	0.25–0.50	< 0.25
Tailings unit weight	> 2.00	1.70–2.00	1.40–1.70	< 1.40
Corrosiveness of tailings	< 0.25	0.25–0.50	0.50–0.75	> 0.75
Closure design	> 0.75	0.50–0.75	0.25–0.50	< 0.25
Knowing the hazards and risks of the tailings dam before closing	> 0.75	0.50–0.75	0.25–0.50	< 0.25
Slope ratio of tailings dam (1:n)	< 1.00	1.00–3.00	3.00–5.00	> 5.00
Dam layout	> 0.75	0.50–0.75	0.25–0.50	< 0.25
Dam break	0	–	–	1
Leakage damage	0	–	–	1
Overtopping	0	–	–	1
